# Should we consider gender aspects in pharmacology teaching? No—we must!

**DOI:** 10.1007/s00210-025-04782-9

**Published:** 2025-11-08

**Authors:** Jan Matthes

**Affiliations:** Centre of Pharmacology, Department II, and Medical Faculty, Student Dean’s Office, University of Cologne and University Hospital Cologne, Cologne, Germany

**Keywords:** Medical education, Gender, Pharmacology teaching

## Abstract

Gender aspects must be part of pharmacology teaching. Pharmacology offers a wealth of examples that prove on the one hand that sex and gender aspects of drug therapy are clinically relevant and on the other hand show that there are still striking omissions in pharmacological research and drug approval. And we should not forget that drug therapy is more than just applied pharmacology and includes, for example doctor-patient communication in the context of a prescription conversation. Moreover, it is not only that sex can make differences in pharmacodynamics and pharmacokinetics and thus in drug therapy—sex and gender can also play a role in (medical) education. Finally, gender-related discrimination continues to be a problem in our society and universities have set themselves the task of having an impact on society as the so-called “third mission”. Pharmacology and drug therapy are excellent for working not only on this mission but also on creating awareness among future physicians of the importance of fair and appropriate consideration of sex and gender in medicine and society.

This special issue of the *Naunyn Schmiedeberg’s Archives of Pharmacology* is an important contribution to an essential aspect of science in general and pharmacological research in particular. Furthermore, the role of sex and gender in the daily practice of physicians, including drug treatment, is becoming increasingly important. It should not be neglected that students must be specifically and professionally trained in this regard. In my view, pharmacology teaching can and must play a key role here. I will try to illustrate this below. First, I will refer to the medical aspects and thus to topics to be covered in our teaching. I will begin with examples that illustrate the relationship between biological sex and pharmacology. Then I will discuss aspects of drug therapy that require consideration of the broader concept of gender. In a third section, I will address didactics, as sex and gender also play a role in how we learn and teach. To better understand my perspective, it is perhaps helpful to mention that I am male, work in Germany, am a doctor and am mainly involved in the education of medical students.

## The subject-matter perspective

### Pharmacology and drug effects

*Which statement about primary prevention with medication in coronary artery disease (CAD) is correct? Randomised controlled trials (RCTs)…*

(A) *…prove that aspirin saves lives.*
(B) *…prove that statins reduce the rate of heart attacks.*
(C) *…do not allow a reliable statement about prognostic benefits for women.*

In fact, the first two statements, in the undifferentiated strictness with which they appear here, can at least be discussed, and this is explained by the third statement, which is true. It has been shown that in clinical trials women are often underrepresented. In RCTs on CAD, merely one third of the included patients were female, although a good half of CAD patients are women (Melloni et al. 2010; Gong et al. 2019). In fact, results from studies and meta-analyses are ambiguous and thus Cangemi et al. concluded that “available evidence does not allow to draw strong recommendations for primary CVD prevention” (Cangemi et al. 2017; Mauvais-Jarvis et al. 2021). How does this example relate to pharmacological teaching? We cannot expect (medical) students to question on their own initiative whether and what role gender plays in drug therapy or even regarding pharmacological findings—especially since these aspects are hardly or not at all addressed in current textbooks (see below). Since we are preparing students for lifelong learning, it appears to be important to use examples to illustrate where medical findings need to be questioned rather than making seemingly unambiguous statements. As we teach the principle of weighing up the benefits and harms of a drug, we also need to teach students to evaluate the conclusiveness and soundness of underlying information. This includes the fact that in many cases it is not possible to make a clear, gender-specific statement on drug properties or benefits and harms of drug treatment. On the other hand, there are well-known differences between women and men with respect to pharmacology. In fact, even in basic pharmacology, sex is an important issue. In animal research, sex has often not been considered at all and in studies considering animal sex most often males were used exclusively (Beery and Zucker 2011; Mamlouk et al. 2020). Since (eucaryotic) cell lines are derived from individuals with a certain sex even these cell lines have a sex which could have implications for experiments on drug metabolism, for example (Shah et al. 2014; Madla et al. 2021). As pharmacology teachers, we should be aware of sex bias in preclinical and clinical research, as well as—no less importantly—the often lack of knowledge about possible sex differences. And we should tell students about the resulting uncertainty instead of pretending that there are no sex-related differences and thus male and female patients can be treated the same. Because we cannot specifically address gender dependencies on every topic, we may have to do so by way of example: differences between women and men in drug metabolism via the enzyme CYP2D6 are associated with differences in the occurrence of adverse effects of drugs that are CYP2D6 substrates (Thürmann et al. 2006). Furthermore, for the desired effects of antihypertensive drugs, differences between male and female patients have been reported. For example, amlodipine appeared to lower blood pressure more effectively in females (Kloner et al. 1996). Both, pharmacokinetic (metabolism via CYP3A4) and pharmacodynamic mechanisms (different responsiveness of blood vessels) have been discussed to explain this finding. Thus, CYP2D6-mediated metabolism and the antihypertensive effect of amlodipine can be used as examples of clinically relevant differences depending on a patient’s sex and linked to sex-dependent underlying mechanisms.

### Implementation of drug treatment

*Which of the following combinations for counselling and treatment is most likely to benefit patients?*

(A) *Female physician, female patient*
(B) *Female physician, male patient*
(C) *Male physician, female patient*
(D) *Male physician, male patient*

To be honest, we cannot be sure (thus—and for other reasons—I would not recommend using the single-choice format here). However, some studies suggest that female patients counselled and treated by female physicians benefit most, e.g. with respect to cardiovascular risk-factor control and diabetes treatment (Schmittdiel et al. 2009). At this point at the latest, we must admit that we cannot explain such findings by classifying patients according to their biological sex only. Even for the definition of biological sex, the classification into female, male and intersex falls short, as for example hormonal fluctuations, age-related changes and anatomical variance are not adequately taken into account. The concept of gender (also) considers socio-cultural aspects, resulting in a much more complex construct. According to Becher and Oertelt-Prigione, the National Institutes of Health (NIH) describe gender as socially constructed and enacted roles and behaviours that occur in a historical and cultural context, varying from society to society and over time (Becher and Oertelt-Prigione 2023). Globalization also plays a role in this context, as we increasingly have patients (but also students) from different ethnic backgrounds. Although at first glance gender as defined by the NIH does not appear to be a pharmacological issue, it is clear that differences in the implementation of guideline recommendations or with regard to medication adherence cannot be adequately explained by biological sex and that gender aspects must be considered here (Gouni-Berthold and Berthold 2013; Lau et al. 2021; Mauvais-Jarvis et al. 2021). Regarding the role of physicians’ gender in differences in drug therapy, differences in communication style have been discussed (Gouni-Berthold and Berthold 2013). For example, female physicians appear to use shared decision-making more often (Cooper-Patrick et al. 1999), a communication approach that was associated with improved medication adherence and subsequently treatment success, e.g. in diabetes (Parchman et al. 2010; Matthes and Albus 2014; Hauser et al. 2015). On the other hand, a patient’s gender appears to have an impact, too, as indicated at the beginning of this section (Schmittdiel et al. 2009; Sandhu et al. 2009). Taken together, doctor-patient communication plays a significant role with respect to gender-related implementation and outcome of drug therapy. As pharmacology teachers, we have the opportunity and, in my view, the duty to enable medical students not only to consider gender-dependent influences in order to choose an effective and safe drug therapy, but also to ensure that this drug therapy is feasible and implemented. Fortunately, intensive efforts have been made in recent years to improve the ability of medical students to prescribe medicines safely (Maxwell et al. 2017; Donker et al. 2022). In addition, we should place even greater emphasis on doctor-patient communication with regard to conducting prescription talks (Hauser et al. 2017; Kirsch and Matthes 2021)—against the background of the complexity of drugs and their properties, something for which pharmacology teachers are predestined.

To summarize this pharmacological perspective: within our discipline, research and teaching cover everything from molecular mechanisms to the clinical application of drugs, which predestines pharmacology to reflect and link the significance of sex and gender at various levels.

## The educational perspective

Last but not least, I would like to discuss whether and how the sex and gender of students should be taken into account in our teaching and, on the other hand, whether teaching can have an influence on gender sensitivity. The question may arise as to whether the learning behaviour and abilities of male and female students differ and whether one should therefore choose, for example gender-adapted teaching–learning formats. In fact, the “gender difference hypothesis” is still discussed in psychology, although research broadly supports the “gender similarity hypothesis”, i.e. males and females are much more likely to have similar abilities than to be divided in terms of capacity (Hyde 2014; Gill 2020). Even persistent attributions such as better mathematical abilities of men cannot be upheld against the background of current studies or—as in the case of the supposedly better spatial imagination—are more likely to be due to unequal treatment in school education or gender gaps in out-of-school experiences. On the other hand, we in fact see gender differences in medical career considerations (Burgos and Josephson 2014; Alers et al. 2014). Analyses of laureates of the German Pharmacological Society and authorships in *Naunyn–Schmiedeberg’s Archives of Pharmacology* suggest a gender gap in the field of pharmacology as well, both on the national and international level (Zehetbauer et al. 2022; Halling et al. 2025). In summary, aspects of learning psychology and capacity play a negligible role at best, but the same gender aspects that are important in society per se also seem to be important in (medical) education and professional career. It is all the more interesting that medical education can influence the attitude and awareness of students. In a study at four medical faculties in Germany, Laura Wortmann and colleagues showed that “implementation of gender medicine in the medical curriculum, attending courses on gender education as well as one’s gender and interest [had] a significant impact on medical students’ gender competencies” (Wortmann et al. 2023). This shows that gender-sensitive teaching per se can have an influence on the gender competencies of medical students. Given these findings and the pharmacological issues discussed above, it is all the more surprising that sex and gender are given too little or no consideration in medical (including pharmacology!) textbooks (Dijkstra et al. 2008) or that there is even a clear gender bias (Parker et al. 2017). In pharmacology teaching, we can already contribute to greater awareness of gender issues with little effort, e.g. by not predominantly using cases of male patients in lectures or small group lessons and, where possible, even choosing case examples in which the patient’s gender actually plays a (clinically) relevant role. With respect to Schwab’s concept of “curriculum deliberation”, Bordage and Harris recommended involving those “who ‘live in’ or are strongly affected by the education programmes” into curriculum development, i.e. to involve students (Bordage and Harris 2011). An own study showed that medical students indeed can make interesting contributions to the development of their curriculum (and I am not only writing this because they attached great importance to pharmacology) (Dafsari et al. 2017). A survey of medical students in the USA showed that they were able to differentiate between sex- and gender-sensitive medicine and, for example women’s health, and that they perceived that their curriculum was predominantly related to males (Jenkins et al. 2016). Well over 90% of respondents agreed that knowledge of sex- and gender-sensitive medicine improves the ability to treat patients and should be part of the medical school curriculum. In my view, this means that we do not have to be prepared for resistance from students if we were to add gender aspects to the already challenging pharmacology education, but on the contrary would probably knock on open doors. Given the thematic focus of a special issue like this within a pharmacological journal, I will avoid a digression on the effects of gender-sensitive language (Sczesny et al. 2016). However, my expectation would be that—especially in the German-speaking countries where I work—the use of gender-sensitive formulations would help to always ask ourselves whether the aspect we are talking about has gender-dependent facets or whether we don’t even know that.

## Summary and conclusion

In summary, I strongly urge that we consider gender aspects in pharmacological teaching. It is unscientific and possibly disadvantageous for patients if we ignore known influences of sex and gender on drug therapy or ignore the fact that not enough is known about such influences. Pharmacology offers a wealth of examples that prove on the one hand that sex and gender aspects of drug therapy are clinically relevant and on the other hand show that there are still striking omissions in pharmacological research and drug approval. Furthermore, we should not forget that drug therapy is more than just applied pharmacology and includes, for example doctor-patient communication in the context of a prescription conversation. Moreover, sex can not only cause differences in pharmacodynamics and pharmacokinetics, and gender can affect drug therapy even beyond that—sex and gender can also play a role in (medical) education. Finally, gender discrimination continues to be a problem in our society and universities have set themselves the task of having an impact on society as the so-called “third mission”. Pharmacology and drug therapy are excellent for working not only on this mission but also for creating awareness among future physicians of the importance of fair and appropriate consideration of gender in medicine and society.

This leads me to my final question and the title of this article already spoiled what I think the answer should (must!) be…

*Should we consider gender aspects in pharmacology teaching?*

(A) *Never ever.*
(B) *Maybe later.*
(C) *Yes, we should.*
(D) *No – we must!*

## Data Availability

No datasets were generated or analysed during the current study.
